# Exotic Mammals Disperse Exotic Fungi That Promote Invasion by Exotic Trees

**DOI:** 10.1371/journal.pone.0066832

**Published:** 2013-06-24

**Authors:** Martin A. Nuñez, Jeremy Hayward, Thomas R. Horton, Guillermo C. Amico, Romina D. Dimarco, M. Noelia Barrios-Garcia, Daniel Simberloff

**Affiliations:** 1 Department of Ecology and Evolutionary Biology, University of Tennessee, Knoxville, Tennessee, United States of America; 2 Laboratorio Ecotono, INIBIOMA, CONICET-Universidad Nacional del Comahue, Bariloche, Río Negro, Argentina; 3 Department of Environmental and Forest Biology, State University of New York - College of Environmental Science and Forestry, Syracuse, New York, United States of America; Institute of Botany, Czech Academy of Sciences, Czech Republic

## Abstract

Biological invasions are often complex phenomena because many factors influence their outcome. One key aspect is how non-natives interact with the local biota. Interaction with local species may be especially important for exotic species that require an obligatory mutualist, such as Pinaceae species that need ectomycorrhizal (EM) fungi. EM fungi and seeds of Pinaceae disperse independently, so they may use different vectors. We studied the role of exotic mammals as dispersal agents of EM fungi on Isla Victoria, Argentina, where many Pinaceae species have been introduced. Only a few of these tree species have become invasive, and they are found in high densities only near plantations, partly because these Pinaceae trees lack proper EM fungi when their seeds land far from plantations. Native mammals (a dwarf deer and rodents) are rare around plantations and do not appear to play a role in these invasions. With greenhouse experiments using animal feces as inoculum, plus observational and molecular studies, we found that wild boar and deer, both non-native, are dispersing EM fungi. Approximately 30% of the Pinaceae seedlings growing with feces of wild boar and 15% of the seedlings growing with deer feces were colonized by non-native EM fungi. Seedlings growing in control pots were not colonized by EM fungi. We found a low diversity of fungi colonizing the seedlings, with the hypogeous *Rhizopogon* as the most abundant genus. Wild boar, a recent introduction to the island, appear to be the main animal dispersing the fungi and may be playing a key role in facilitating the invasion of pine trees and even triggering their spread. These results show that interactions among non-natives help explain pine invasions in our study area.

## Introduction

Biological invasions are complex phenomena because many factors influence their outcome. One key aspect is the interaction of non-natives with the local biota [1]. Interaction with local species (native or non-native) are important for all species but may be especially important for the successful invasion of exotic species that require an obligatory mutualism, as is the case for members of Pinaceae [2]. Most Pinaceae do not need biotic vectors for seed and pollen dispersal [3]. However, all Pinaceae require ectomycorrhizal (EM) fungi to thrive.

Members of the Pinaceae are compatible with a wide variety of EM fungi in their native ranges [4] but are often incompatible with EM fungi native to areas outside of the biogeographic range of the Pinaceae family [5], [6]. Within the native ranges, where EM fungi can be widespread and where dense spore banks may be common, Pinaceae species are probably rarely limited by EM inoculum. Outside their native ranges, Pinaceae-compatible EM inoculum may be rare or absent. EM fungal species disperse poorly from exotic Pinaceae plantations in several areas, including New Zealand, Australia, and North America (in areas with no native pines) [7]–[9]. When compatible EM inoculum is not abundant, failure to locate EM mutualists can limit the ability of Pinaceae species to invade. We have previously shown that Pinaceae invasions on Isla Victoria, Argentina, are limited by their ability to locate compatible EM inoculum [10], suggesting that these invasions are essentially co-invasions (i.e., the trees can form EM only with fungi from their native range) [11]. Pinaceae invasion is a current environmental problem in many parts of the world, especially in the southern hemisphere, implying that at least some Pinaceae-compatible EM inoculum can disperse with Pinaceae species, perhaps when the trees are introduced to new geographic regions, facilitating co-invasions of host trees and the mycorrhizal fungi associated with them [12]–[14].

EM fungi and seeds of Pinaceae disperse independently, so different vectors can disperse them [15], [16]. EM fungi in their native ranges are known to be dispersed by wind [17] and mammalian vectors [18] and may be dispersed by other vectors such as invertebrates [19] and water [20]. Wind is the main disperser of seeds of many Pinaceae species and has been suggested as the main agent dispersing fungal spores. However, wind is expected to produce a diffuse spore rain at long distances [21]. When spore sources are not dense or prolific, this diffuse spore rain may not allow sufficient accumulation of spores to inoculate compatible plants. This problem can be especially important in the non-native ranges of the fungi, where Pinaceae have limited sources of compatible propagules. Biotic vectors may also be important. For example, mammals such as deer, wild boar, and rodents can disperse EM fungi in their native ranges through their feces [18], [22], and mammal feces can contain high spore densities (up to 10^8^ spores/g in deer feces [18]).

Pinaceae species have been introduced mainly for forestry purposes in many different areas of the world where they naturalize and invade [12], [23], [24]. Pines are among the most successful groups of invasive plant species [25]; however, they also fail to invade in many different locations [26]. It has been proposed that biotic filters can operate to cause unsuccessful invasions [14]. As stated above, one factor that can be important in the extent of colonization by Pinaceae is the dispersal of their mutualistic EM fungi [10], [27]. The goal of this study is to analyze the role and nature of EM fungal dispersal by local mammals in Pinaceae invasions on Isla Victoria, a 20 - km long island in Nahuel Huapi National Park, Argentina. We specifically ask: which mammalian vectors play a role in the dispersal of exotic EM fungi on Isla Victoria, and what fungal species are being dispersed? Approximately 135 species of exotic trees, many of them Pinaceae, were introduced to different parts of Isla Victoria, especially in the central area, between 1910 and the late 1930s. Several of the non-native tree species have begun colonizing native forest (dominated by the natives *Austrocedrus chilensis* and *Nothofagus dombeyi* [28], [29]), but all are limited to areas near the original plantations. Far from plantations Pinaceae are limited, in part, by lack of compatible EM fungi [10]. Among the species able to colonize the forest outside of the plantations are *Pseudotsuga menziesii* and several *Pinus* spp., which are obligatorily ectomycorrhizal and the focus of this study.

## Methods

From observations in the area and previous studies on spore dispersal by mammals, we identified three groups of species (deer, boar, and rodents) as potential dispersal vectors. Red deer (*Cervus elaphus*) and fallow deer (*Dama dama*) are Old World species that have long been present and are ubiquitous on the island, and deer have been found to disperse viable mycorrhizal spores in the native range of *Pinus* [18]. Wild boar (*Sus scrofa*) invaded the island in 1999 and have recently achieved high densities. We have frequently observed wild boar in exotic tree plantations where they consume hypogeous (i.e. fruiting belowground) fungi, as they do in their native ranges [30]. Because mice have been shown to be efficient vectors for EM fungi and they are native to the island, we had intended to include mice in this investigation. However, mice are extremely rare near plantations, and we were unable to capture enough mice for this study (see results section). All the other native mammals that could disperse fungi are very rare in the areas where plantations and invasions occur (park ranger Damian Mujica, pers. comm. [31]). Based on this information, we inferred that the most likely candidates to consume and disperse exotic EM fungi are deer and wild boar. Squirrels, which are common vectors of mycorrhizal spores in the native ranges of the introduced trees [32], [33], are not native and are not found in the region.

### Mouse Capture

To assess if a native rodents could disperse EM fungi, we conducted three campaigns to trap mice and collect mice feces: two in fall, and one in spring. We set up 100 Sherman traps in areas adjacent to the plantations (from 0 to 200 meters) for three consecutive days (300 trap/day per campaign, total 900 trap/days). The traps were placed in the ground, separated by 10 meters from each other, and baited with peanut butter and oatmeal. Traps were checked twice daily, once in the morning and once in the evening. When mice were captured, they were released immediately after we detected their presence, and we searched for feces inside the traps. All animal capturing and handling procedures followed the guidelines of the American Society of Mammalogists [34]. Our methods were also approved by the authorities of the Nahuel Huapi National Park.

### EM Fungal Dispersal by Mammals

During peak fungal fruiting periods (austral fall and spring 2008), fresh deer and wild boar feces were collected in native forests near plantations (less than 200 meters from plantations), where chances of finding dispersal of fungi associated with plantation trees are greatest. We collected feces haphazardly from areas surrounding plantations in the study site (see map in Simberloff et al. 2002). We collected up to 20 deer pellets and entire wild boar individual fecal piles. We bagged each sample independently to avoid contamination. Fecal pellets were air-dried and stored for bioassay. To reduce the chances of contamination produced after the feces were deposited by the animals, we used sandpaper to remove the outermost 1 mm of fecal material from each collection used for the inoculation.

We used these feces in an experiment in which we planted surface-sterilized seeds of *Pseudotsuga menziesii* or *Pinus ponderosa*, which are two of the most planted species and commonly found outside the plantations on the island; *P. menziesii* is the most invasive Pinaceae in the area [28]. For each species, we planted three seeds in each of 160 pots in a factorial design with 40 pots per treatment and the following variables: feces identity (deer or boar), and seed species (*P. menziesii* or *P. ponderosa*). We had 40 additional pots, 20 for each plant species, half with sterilized wild boar feces and half with sterilized deer feces, for a total of 200 pots in the experiment.

Only the first seed to germinate in each pot was allowed to grow. Some species of fungi form ectomycorrhizas only with individuals of the genus *Pinus* or only with individuals of the genus *Pseudotsuga*, so by having specimens of both tree genera we expected to capture a representative sample of the fungi dispersed. Seeds were planted in pots in a greenhouse in sterile soil (autoclaved twice) from the studied area. To sterilize soil, we autoclaved it at 121°C for 20 minutes, waited 24 hours, then autoclaved it at 121°C for a further 20 minutes. To each pot we added 5 ml of dry, coarsely ground (particles size up to ca. 2 mm) fecal material. We added feces instead of fecal extracts to mimic as nearly as possible the results of seeds landing on or near the feces. As a control we used surface-sterilized seeds growing in sterile soil with double-autoclaved ground fecal pellets to detect greenhouse EM contamination as in previous studies.

We harvested seedlings after 9 months and recorded the colonization of EM species in each seedling (presence of EM root tips and percent of root tips colonized). We randomly collected 5 colonized root tips per seedling. Colonized root tips were collected and stored in 2X CTAB buffer solution [35], and specimens of EM fungi were identified using molecular methods. All necessary permits were obtained from the authorities of the Nahuel Huapi National Park for the described field studies.

### Molecular Methods

We extracted DNA from whole root tips stored in 2x CTAB buffer using the protocol of Hayward and Horton (2012), but using 6M guanidine hydrochloride (Qiagen Buffer PB; Qiagen inc., Valencia, CA) instead of NaI as the chaotropic salt to bind to DNA silica. We amplified the nuclear ribosomal internal transcribed spacer region 1, 5.8s ribosomal subunit gene, and the internal transcribed spacer region 2 from root tips using combinations of the following primers: ITS1, [36] ITS1f, [37] and NSA1 [28] (Forward primers), and ITS4, [26] NLB4 [38] (reverse primers). PCR conditions were as follows: 3 minutes at 94°C, followed by 35 cycles of 35 seconds at 94°C, 55 seconds at 53°C, and 45 seconds at 72°C, adding 2 seconds per cycle to the extension time, with a final extension period of 10 minutes at 72°C. We generated restriction fragment length polymorphism (RFLP) profiles from the amplicons using the restriction enzymes *Hinf*I and *Hae*III (New England Biolabs, Ipswich, Massachusetts) following the manufacturer’s guidelines. We sequenced 2–3 exemplars of each amplicon displaying a unique RFLP pattern using PCR primers on an ABI 3730xl sequencer (Life Technologies Inc., Carlsbad, CA).

We aligned all sequences using MUSCLE [39] as implemented in Seaview 4.3.0 [40]. We used MOTHUR 1.25.1 [41] to create strict consensus sequences for operational taxonomic units (OTUs) grouped at the 97% sequence similarity level based on furthest-neighbor clustering, counting indel gaps as a single character and not counting beginning and ending gaps. We compared these consensus sequences to GenBank deposited sequences using Blastn to assign OTUs to known taxa. OTUs were considered conspecific with identified taxa in GenBank at or above 97% ITS similarity. We used consensus sequences because they are more conservative in matching reference sequences [42]; polymorphisms observed in the study (which will be represented in the query sequence by IUPAC ambiguity codes) cannot match isolate sequences in GenBank.

## Results

Boar and deer appear to disperse EM spores efficiently, with many feces containing spores. Approximately 15% of the seedlings inoculated with deer feces were colonized by EM fungi, while approximately 30% of the seedlings inoculated with wild boar feces were EM (Table 1). Boar feces were more likely to inoculate seedlings than deer feces (Chi-square = 6.13, DF = 1, P = 0.013). None of the seedlings in the control (sterile) treatment were colonized. There were no clear differences in colonization rates between *Pinus* and *Pseudotsuga* (Chi-square  = 0.62, DF = 1, P = 0.43).

**Table 1:** Percent of seedlings colonized when grown with feces of deer and wild boar.

Percent seedlings colonized by EM fungi (including dead seedlings as non-mycorrhizal for a more conservative estimation of animal dispersal)	Boar	Deer
*Pinus ponderosa*	32.5	17.5
*Pseudotsuga menziesii*	37.5	15
**Percent colonized (not including dead seedlings)**	Boar	Deer
*Pinus ponderosa*	36.11	20.59
*Pseudotsuga menziesii*	46.87	21.43

Some seedlings died during the experiment, and we present here the data with and without the dead seedlings. Data treating dead seedlings as non-mycorrhizal give a more conservative estimate of the dispersal by mammals. The total number of seedlings per treatment was 40.

We deposited representative sequences for each operational taxonomic unit generated in this study in GenBank under accession numbers KC179047 to KC179053. We detected only seven EM fungal species colonizing seedlings: an *Amphinema* species, a *Melanogaster* species, *Rhizopogon cf. rogersii, Rhizopogon cf. arctostaphyli, Rhizopogon roseolus, Suillus luteus* and *Hebeloma mesophaeum* (Table 2). All these species are non-native to the study region and have been described previously from the native range of the Pinaceae [43]–[45]. Based on results from Table 2 there were no noticeable differences in the composition of the mycorrhizal species dispersed by deer or wild boar, with all species detected on more than one seedling being transported by both deer and boar. Approximately 70% of identified fungi belonged to the genus *Rhizopogon*, a genus that forms mycorrhizae almost exclusively with Pinaceae.

**Table 2:** Fungal species identified on the seedling root tips, their host tree, and animal vector.

Fungal species	Host tree	Deer	Boar
*Amphinema* sp.	*Pinus ponderosa*	1	0
*Hebeloma mesophaeum*	*Pseudotsuga menziesii* and *Pinus ponderosa*	4	3
*Melanogaster* sp.	*Pinus ponderosa*	0	1
*Rhizopogon cf. arctostaphyli*	*Pinus ponderosa*	3	9
*Rhizopogon cf. rogersii*	*Pseudotsuga menziesii* and *Pinus ponderosa*	12	11
*Rhizopogon roseolus*	*Pinus ponderosa*	1	2
*Suillus luteus*	*Pinus ponderosa*	1	4

Numbers in the table indicate the number of seedlings on which these fungi were found. It was impossible to obtain DNA amplifications on some colonized root tips, so these data are not representative of the overall dispersal by the different mammals.

Mice are very rare near the plantations on Isla Victoria. In the course of three campaigns we captured only one individual mouse (*Oligoryzomys longicaudatus*, Cricetidae). In a nearby forest on the mainland (Llao Llao forest, 1 km from the island), the same crew leader (G. Amico) using the same method (e.g., same bait, same strategy to place traps) captured approximately 26.7 mice per day. We therefore suggest that the role of mice in dispersing non-native EM inoculum is probably negligible around the plantations, especially in comparison to those of other non-native mammals.

## Discussion

**Figure 1:**
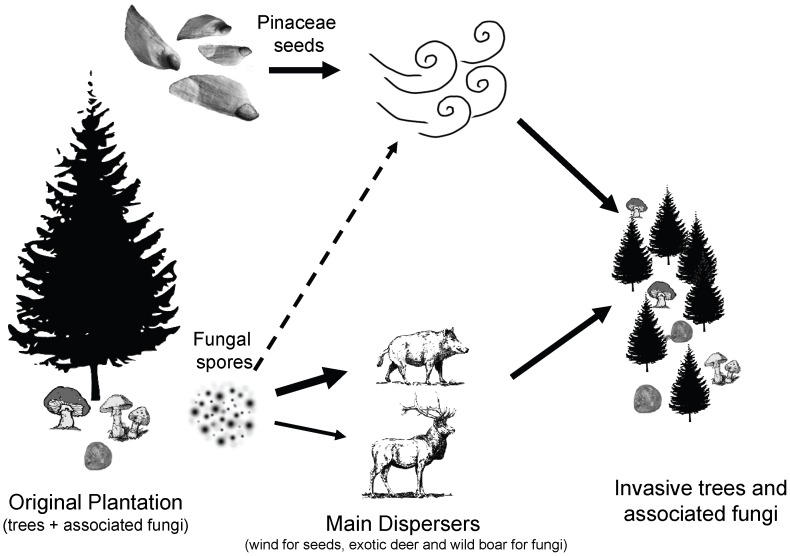
Diagram of the hypothesized process of invasion by Pinaceae and their symbionts, with their main vectors. Our results show that boar play a principal role in the dispersal of EM fungi, and deer may also play an important role. We did not test the role of wind; data are unclear on the role of wind on EM fungi dispersal.

These results suggest that non-native mammals can be important dispersers of non-native EM fungi, which in turn can promote Pinaceae invasion (Fig. 1). Deer and boar may be playing a key role in the co-invasion of Pinaceae and their required mycorrhizal fungi, which is not surprising given that they fulfill the role of spore dispersers in their native ranges, where Pinaceae and these mammals overlap [18], [30].

Old World deer were introduced to the study area at the same time that Pinaceae started to be planted, and deer are known to consume fruiting bodies of both epigeous and hypogeous fungi [18]. One species of native deer could potentially disperse fungal spores. This is the native dwarf deer *Pudu pudu,* but this species has become very rare in the area probably because of human activities [46]. The few individuals present nowadays are found only in the northern portion of the island (park ranger Damian Mujica, pers. comm.), away from our study area.

Wild boar is known to search for hypogeous fungi and disseminate their spores in their native range [30]. Boar are thought to aid the genetic mixing of spatially separated fungus populations [30], and they are also implicated in the spread of soil-borne fungal pathogens [47], [48]. Given the recent introduction of wild boar to Isla Victoria (1999) and the fact that daily movements range from 1–12 km in the boar native range [49], [50] and non-native range [51], [52], it is reasonable to hypothesize that boar will facilitate a progressive build-up of spore banks of EM fungi known to produce resistant propagules [18], [53], [54], allowing an expansion of the invasive range of Pinaceae species.

Different EM fungal species have different dispersal abilities [17]. Our results suggest that members of the genus *Rhizopogon* are the dominant EM fungi dispersed by non-indigenous local mammals. This finding agrees with the previous results of Nuñez et al. [10], who found high levels of *Rhizopogon* colonization near plantations but low levels far from plantations. Suilloid fungi (a group including *Rhizopogon*, *Suillus*, and other genera) are the most commonly observed EM symbionts of invasive Pinaceae species [11], [55]; they also appear to be the most common non-native EM fungi on Isla Victoria both above and below ground (Jeremy Hayward, unpublished data). Suilloid fungi possess resistant spores capable of germinating after years in the ground [53], [56], making them particularly suited to accidental or deliberate co-introduction with EM tree species. It is this same quality that contributes to their accidental spread throughout the Southern Hemisphere with introduced soil and litter [57]–[59].

Rodents can disperse mycorrhizal fungi [32], [33], [60]–[63]. However, data in the study area suggest that rodents are very infrequent near plantations, so their ecological relevance as mycorrhizal vectors in this system may be nil. Also, our previous study on seed predation in this area found notably low levels of seed predation in areas near plantations, a pattern we attributed to low numbers of seed predators, including rodents [64].

Other factors influence pine invasion in the area, such as herbivory [65], [66], climate [14], seed predation [64], propagule pressure [67], and competition with local species [28], [29]. Our findings show that dispersal by non-indigenous mammals may play a role in spore dispersal, assisting the Pinaceae/EM fungal co-invasion. This can be categorized as a case of invasional meltdown [68], [69], where the presence of one exotic species (in this case invasive mammals) exacerbates the impact of other exotic species (non-native EM fungi and Pinaceae).

Wind may also disperse spores across the Patagonian forest; however, the role of wind in dispersing EM inoculum is not clear in these forests. Buller [15] hypothesized that wind is effective in dispersing spores, and this claim is supported by work by James and Vilgalys [70], Aylor [71], and Peay et al. [17], among others. Peay et al. [17] showed that some species of EM inoculum may be wind-dispersed over kilometers across unforested sites but observed strong dispersal limitations. On the other hand, Li [72] and Galante et al. [21] suggest that the majority of spores are deposited in close proximity to the sporocarps, with numbers declining exponentially with distance. Wind is likely playing a role in our studied site, especially for epigeous taxa such as *Suillus* and *Hebeloma*, and more research is clearly needed on this topic.

Hypogeous fungi, the most abundant in our samples, are unlikely to be dispersed by wind as much as epigeous fungi are. For example *Rhizopogon* and *Melanogaster*, two hypogeous groups, were observed on seedlings in the fecal bioassays and are probably not wind-dispersed to any great degree. Peay et al. [17] found two *Rhizopogon* species in their spore traps suggesting some amount of dispersal via wind, but the spores were far less frequently observed than those of ballistosporic taxa that forcibly discharge their spores into the air such as *Cortinarius, Hebeloma, Laccaria, Russula, Suillus, Thelephora* and *Tomentella*. Like other hypogeous fruiters, *Rhizopogon* and *Melanogaster* are statismosporic and emit odors to attract mammals that eat the sporocarps and later deposit millions of spores in fecal pellets. That *Rhizopogon* was so frequently encountered in our fecal bioassays (Table 2) suggests dispersal by non-native mammals is important for these taxa in the study area and may contribute substantially to the co-invasion of the non-native fungi and their associated host trees.
